# Supplementary material for: Properties and efficient scrap-and-build repairing of mechanically sheared 3’ DNA ends
Source: Commun Biol. 2019 Nov 8;2:409. doi: 10.1038/s42003-019-0660-7 (PMC6841706; doi:10.1038/s42003-019-0660-7)
Supplement: Supplementary file 1 — Supplementary Information [file 42003_2019_660_MOESM1_ESM.pdf]

Supplementary Figures

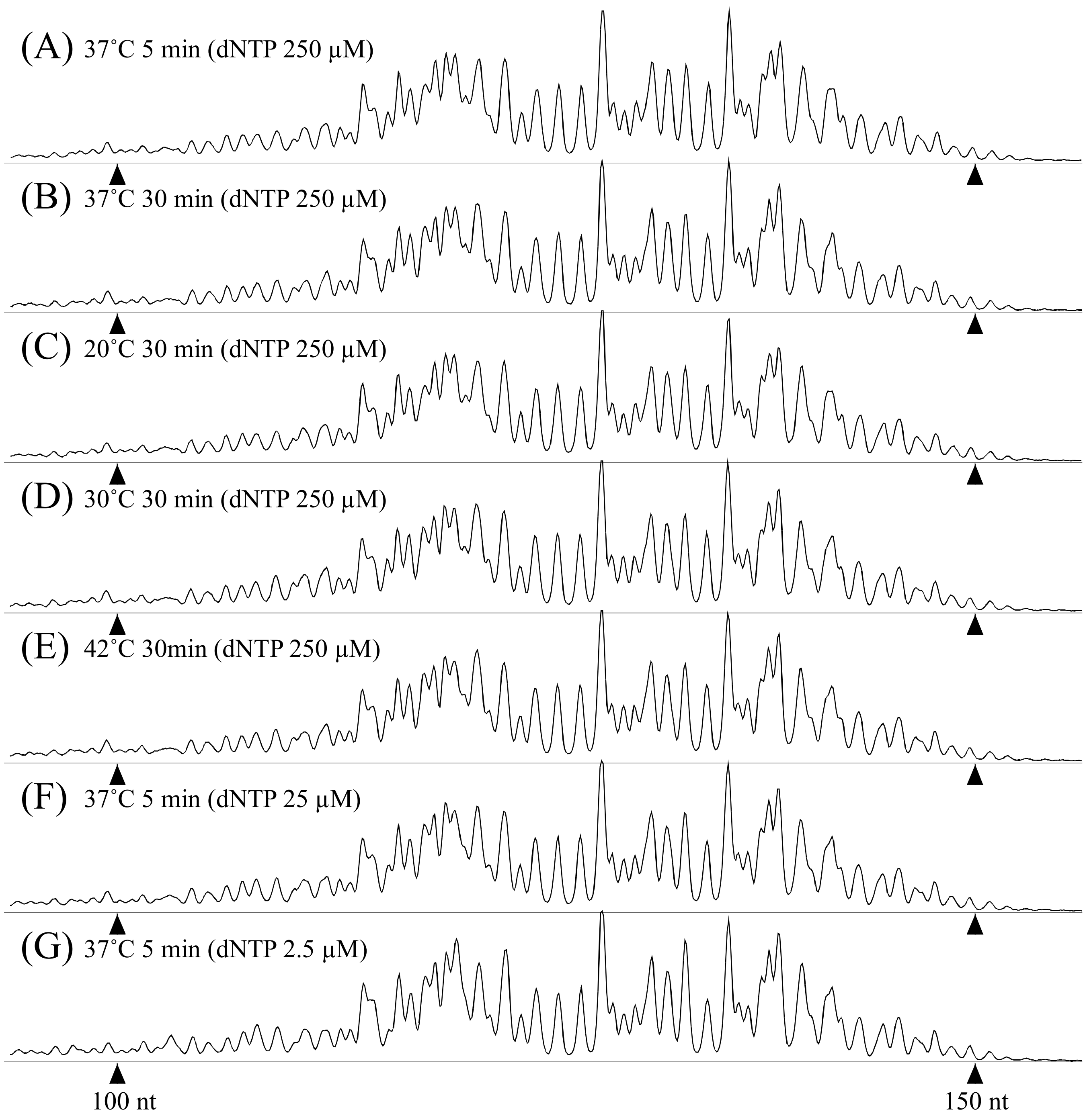

Supplementary Figure 1 **Reaction condition chages of T4DP treatment of H4 mixture had no marked effects.**
H4 fraction DNA mixture was treated with T4DP. Each panel is labeled with incubation temparature, incubation duration, and dNTP concentration. Samples were purified using a DNA clean-up column before mixing with HiDi-LIZ500, and then analyzed by a capillary sequencer. All data were y-axis scaled so that sums of FAM peak areas are apparently even across the panels.

Fig.S1 Y. Ohtsubo et al.

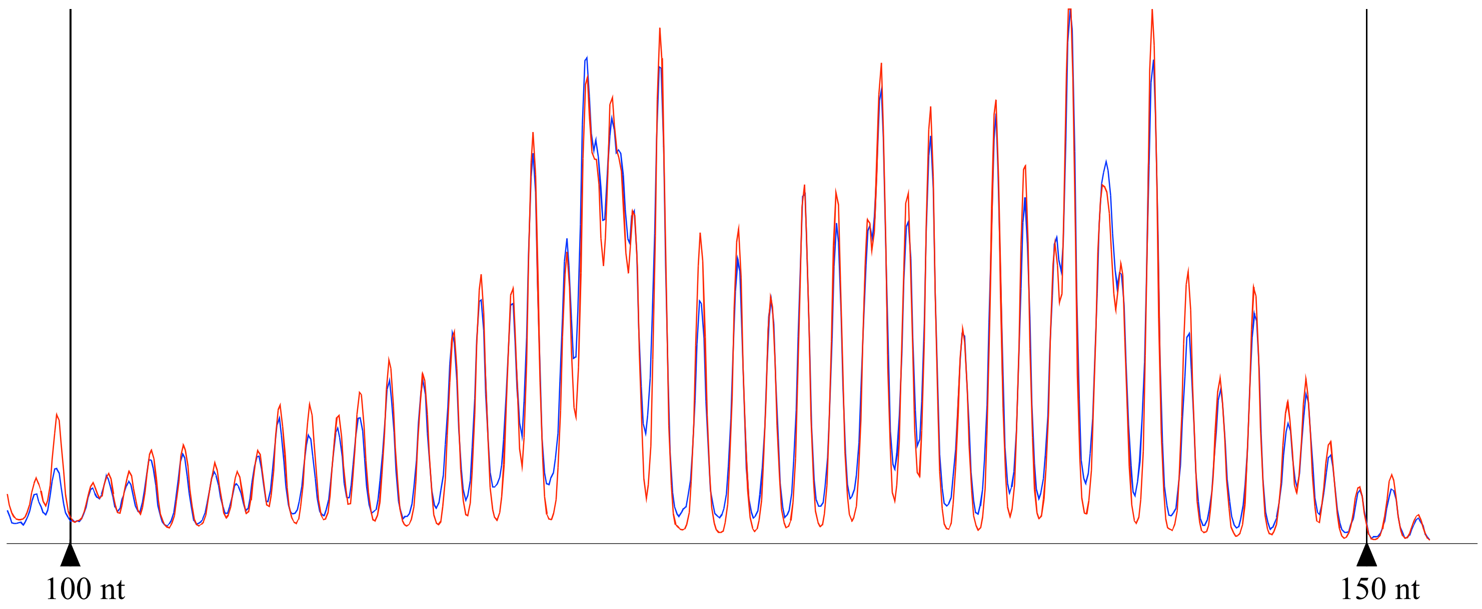

Supplementary Figure 2 **E3T4-T4DP treatment of H4 fraction resulted in sharper peaks.**

Data shown in panels E (H4 fraction DNA treated by SAP-T4DP; blue lines) and G (treated by E3T4-T4DP; red lines) in Fig. 1 were merged. Two data were y-axis scaled so that the sums of FAM peak areas are apparently even.

Fig.S2 Y. Ohtsubo et al.

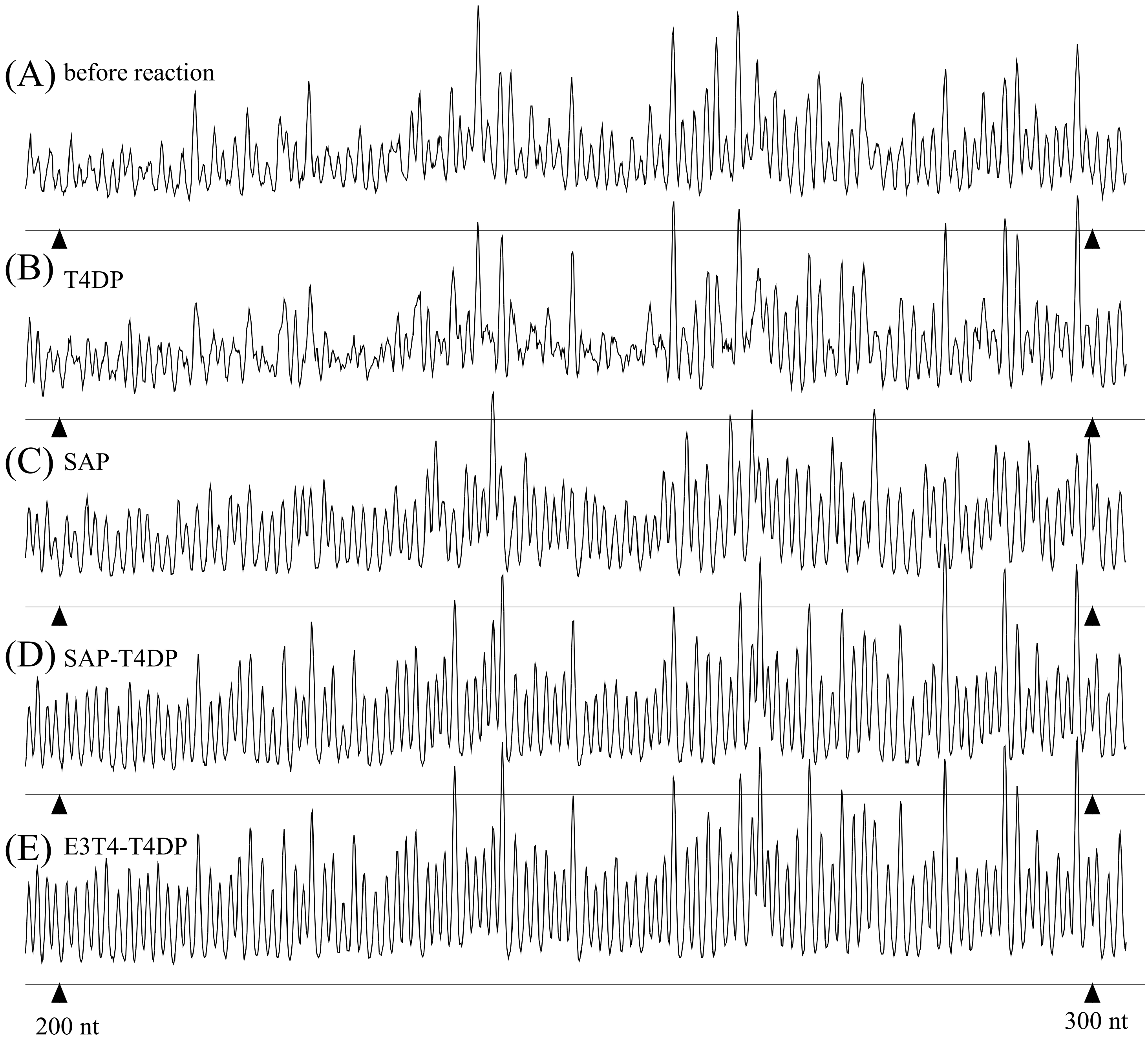

Supplementary Figure 3 **Experiments using DNA termini generated with a mild set of sonication conditions.** The 560-bp DNA was fragmented with a mild set of sonication conditions recommended by the manufacturer to fragment genomic DNAs into 1,500 bp in average. The generated fragments were analyzed by a capillary sequnecer before (A) and after different reactions (B-E). To gain beter resoulution of peaks, injection duration was changed from 90 seconds to 15 seconds.

Fig.S3 Y. Ohtsubo et al.

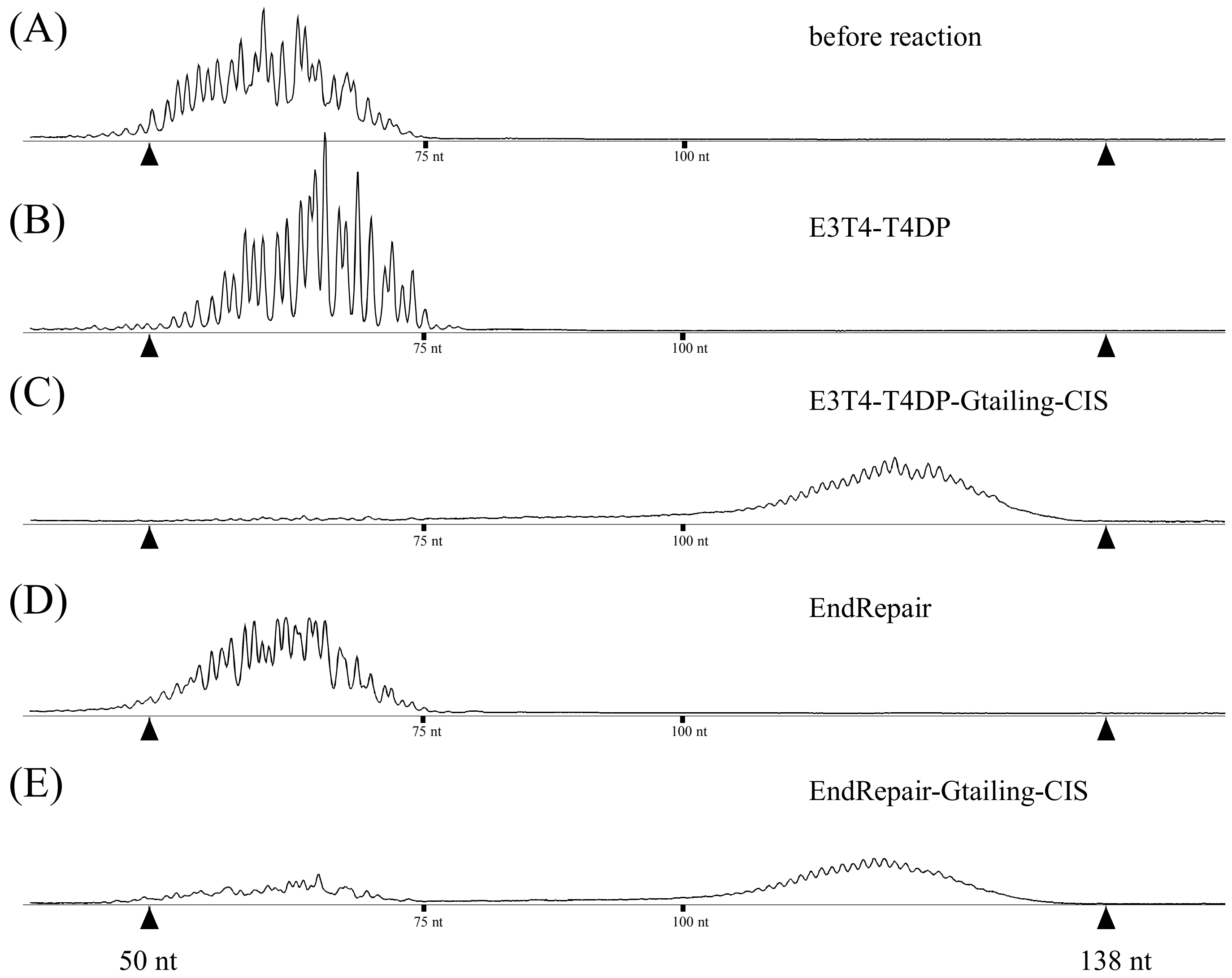

Supplementary Figure 4 **Comparison of SB-repairing and NEB EndRepair kit by using H1 fraction**

(A) H1 fraction DNA before reaction . (B-E) 50 fmol of H1 DNA fraction was subjected to serial reactions as indicated. Between the two enzymatic treatments, and before mixing with HiDi-LIZ500, each samples was purified using a DNA clean-up column. Note that the EndRepair kit resulted in a disorderd pattern (D), but not inefficient CIS reaction (E). T4PNK is possibly contained in the EndRepair kit, and we found that when 5' FAM-labeled DNAs were reacted with T4PNK in the presence of ATP or dNTPs, the reaction products migrated faster (peaks shifted by about 2 nucleotides), and the effect was reverted by SAP treatment (see Supplementary Figure 5 and Supplementary Figure 6). Digestion by an endonuclease (PvuII) of the T4PNK-treated DNA suggested that not 3' but 5' end of the strand, i.e. FAM-moiety, is phsophorylated by T4PNK. As the FAM seems not to be a good substrate of T4PNK, we concluded that incomplete FAM-phospholylation resulted in the disordered pattern, and that the disordered pattern in the panel D does not necessarily indicate inefficiency of the EndRepair kit.

Fig.S4 Y. Ohtsubo et al.

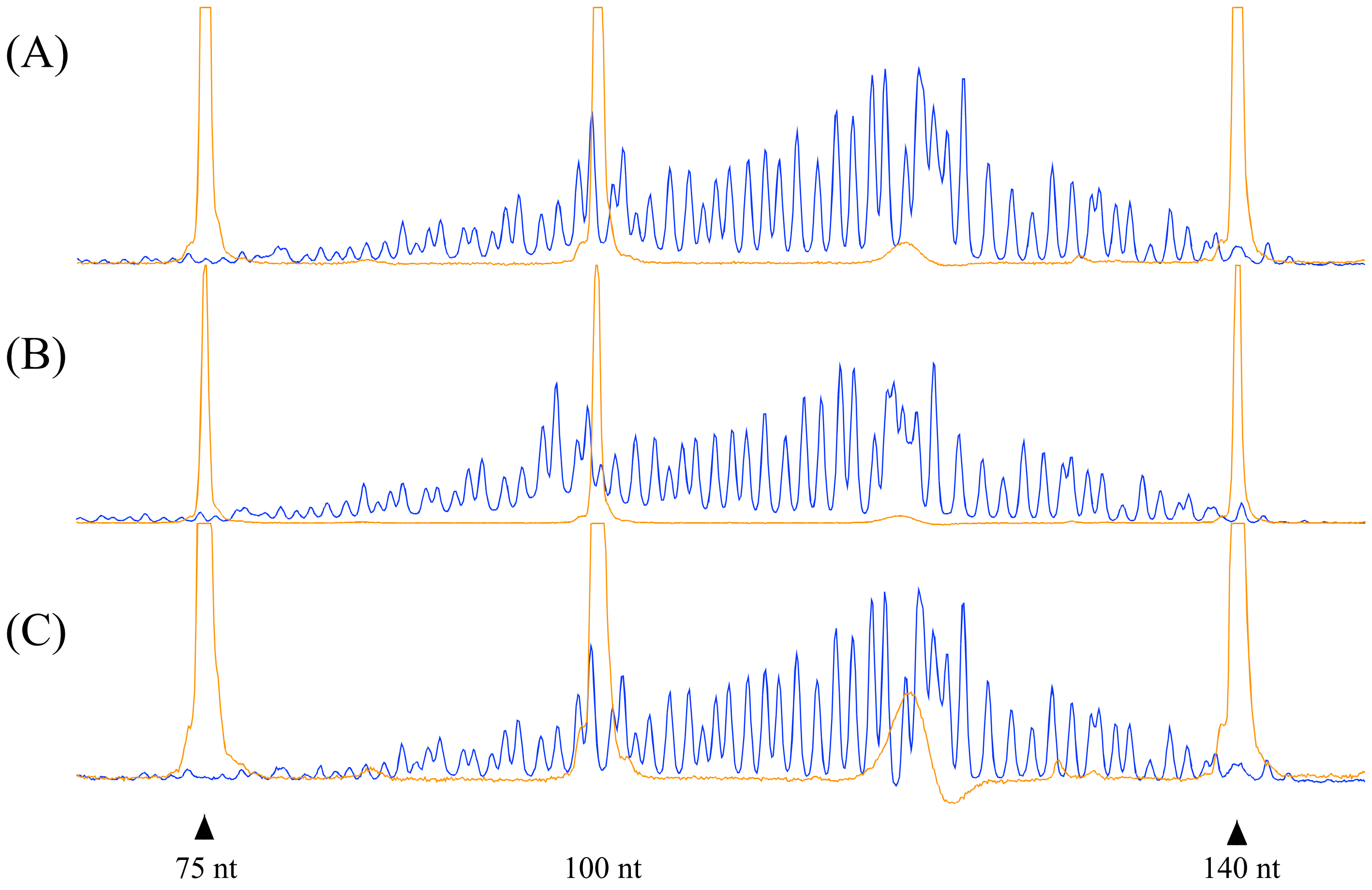

Supplementary Figure 5 **FAM-labeled strand is phosphorylated by T4 polynucleotide kinase.**
The G3 fraction, which was prepared in the same manner as H4 fraction, was subjected for E3T4 treatment for 30 minutes at 37˚C (A) , and then treated with T4PNK in the presence of 1 mM ATP for 1 hour at 37˚C (B), and then treated with SAP for 30 minutes at 37 ˚C (C). After the T4PNK treatment, peaks migrated faster, and the additional incubation with SAP erased the effect, indicating that T4PNK phosphorylates FAM-labeled strand. The site of phosphorylation is most likely the FAM moiety (see Supplementary Figure S6). Samples were purified by using DNA purification column after each treatment.

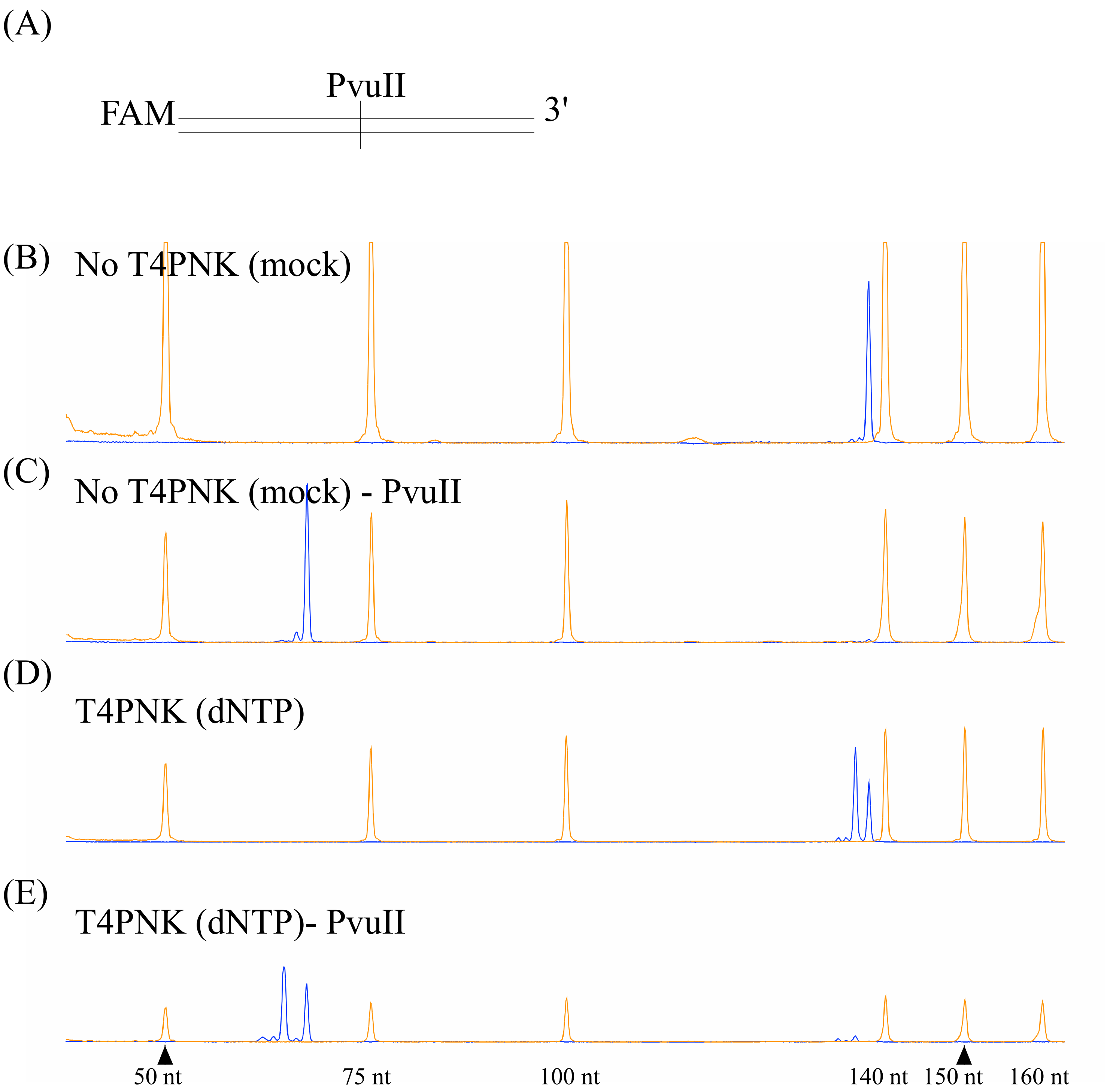

Supplementary Figure 6 **FAM moiety is phosphorylated by T4 polynucleotide kinase (T4PNK) in the presence of dNTP**

(A) Schematic drawing of a140-bp DNA fragment used. This fragment was amplified by KOD DNA polymerase (TOYOBO, Japan), using primers SA560 and SA643 (5'-CTGTCTCCCATTCAGGCTGCGCAACTG-3') and pGiTp as a template. In the midst of the fragment, it carried PvuII recognition site. (B-E) The 140 bp fragment (480 fmol in 10 µl reaction mixture) was incubated for 30 minutes at 37˚C in the absence (B and C) or presence (D and E) of 2.5 unit of T4PNK (TAKARA, Japan) and dNTPs (0.25 mM each), and then analyzed by a capillary sequencer, before (B and D) and after (C and E) PvuII (TAKARA, Japan) digestion. In panel D, in addition to the original peak, a peak that migrated faster appeared suggesting that T4PNK treatment resulted in increase in minus electric charge in a fraction of FMA-labeled strand. The two peaks in panel E after PuvII digestion showed that the site of electric charge increase is not on the 3' end of the FAM-labeled strand, but on the 5' end of the FAM-labeled strand. It is most likely that FAM is phosphorylated by T4PNK. Samples were purified by using DNA purification column after each treatment.
